# White matter hyperintensities segmentation: a new semi-automated method

**DOI:** 10.3389/fnagi.2013.00076

**Published:** 2013-12-02

**Authors:** Mariangela Iorio, Gianfranco Spalletta, Chiara Chiapponi, Giacomo Luccichenti, Claudia Cacciari, Maria D. Orfei, Carlo Caltagirone, Fabrizio Piras

**Affiliations:** ^1^Neuropsychiatry Laboratory, Department of Clinical and Behavioral Neurology, Istituto di Ricovero e Cura a Carattere Scientifico Santa Lucia FoundationRome, Italy; ^2^Department of Radiology, Istituto di Ricovero e Cura a Carattere Scientifico Santa Lucia FoundationRome, Italy; ^3^Department of Neuroscience, Tor Vergata UniversityRome, Italy

**Keywords:** white matter hyperintensities, lesion segmentation, MRI, FLAIR, MCI

## Abstract

White matter hyperintensities (WMH) are brain areas of increased signal on T2-weighted or fluid-attenuated inverse recovery magnetic resonance imaging (MRI) scans. In this study we present a new semi-automated method to measure WMH load that is based on the segmentation of the intensity histogram of fluid-attenuated inversion recovery images. Thirty patients with mild cognitive impairment with variable WMH load were enrolled. The semi-automated WMH segmentation included removal of non-brain tissue, spatial normalization, removal of cerebellum and brain stem, spatial filtering, thresholding to segment probable WMH, manual editing for correction of false positives and negatives, generation of WMH map, and volumetric estimation of the WMH load. Accuracy was quantitatively evaluated by comparing semi-automated and manual WMH segmentations performed by two independent raters. Differences between the two procedures were assessed using Student’s *t*-tests and similarity was evaluated using linear regression model and Dice similarity coefficient (DSC). The volumes of the manual and semi-automated segmentations did not statistically differ (*t*-value = -1.79, DF = 29, *p* = 0.839 for rater 1; *t*-value = 1.113, DF = 29, *p* = 0.2749 for rater 2), were highly correlated [*R*^2^ = 0.921, *F*_(1,29)_ = 155.54, *p* < 0.0001 for rater 1; *R*^2^ = 0.935, *F*_(1,29)_ = 402.709, *p* < 0.0001 for rater 2] and showed a very strong spatial similarity (mean DSC = 0.78, for rater 1 and 0.77 for rater 2). In conclusion, our semi-automated method to measure the load of WMH is highly reliable and could represent a good tool that could be easily implemented in routinely neuroimaging analyses to map clinical consequences of WMH.

## INTRODUCTION

White matter hyperintensities (WMH) are brain areas of increased signal intensity, appearing on T2-weighted (T2-w) or fluid-attenuated inversion-recovery (FLAIR) magnetic resonance imaging (MRI) scans, that result from localized changes in tissue composition (Barkhof and Scheltens, 2002). The etiology of WMH is not specific and may be secondary to ischemia, demyelinating disorders, hydrocephalus, trauma, inflammatory diseases, radiation injury, amyloidosis, and other causes (Pantoni and Garcia, 1997).

WMH are often observed in elderly participants (de Groot et al., 2000a), and their frequency is greater in individuals with cerebrovascular risk factors such as diabetes and hypertension (de Leeuw et al., 2001). Furthermore, WMH are a common finding in those subjects with neurological illnesses, such as stroke (Fazekas et al., 1993), Parkinson’s disease (Marshall et al., 2006), mild cognitive impairment (MCI; DeCarli et al., 2001), Alzheimer’s disease (Fazekas et al., 1996; Hirono et al., 2000), and even in patients with primary mental disorders including mood disorders and schizophrenia spectrum disorders (Brown et al., 1995).

Although neuropathological, clinical, and cognitive significance of WMH is unclear (Gunning-Dixon and Raz, 2000) large epidemiological studies provide evidence that WMH have a strong impact on cognitive functioning (Gunning-Dixon and Raz, 2000) and they have been associated with impairment in a number of domains, including psychomotor speed, frontal executive functions (de Groot et al., 2000b; Prins et al., 2004), and explicit memory (de Groot et al., 2000a).

Interestingly, a systematic review and meta-analysis (Debette and Markus, 2010) provides strong evidence that WMH may be an important predictor of future disease, being associated with an increased risk of stroke, dementia, and mortality. Moreover, it has been shown that WMH often occur in preclinical stages of dementia (such as in MCI patients) and that the presence of WMH may also increase the likelihood of progression from MCI to dementia (Wu et al., 2002).

A challenging issue in studying WMH is their quantification and localization, given their variability and scattered spatial distribution. Two analytic strategies have been used to evaluate WMH on MRI brain images: (1) semi-quantitative rating systems and (2) quantitative volumetric analyses. The semi-quantitative approach mainly consists in the computation of visual ratings and several scales, markedly different in morphological or anatomical definitions, have been proposed and used in literature (Fazekas et al., 1987; Scheltens et al., 1993; Mäntylä et al., 1997; Wahlund et al., 2001). WMH visual rating scales are relatively quick and easy to perform even across scans and scanners, and they are commonly used in clinical and research settings, thus making them attractive for large epidemiological studies. Unfortunately, they have a number of limitations. Indeed, categorical ratings have a restricted range of values that limit the power of association. Moreover, qualitative scales are often subjective in their interpretation, thus limiting inter-rater and intra-rater reliability (Mäntylä et al., 1997) and consistency within longitudinal studies (Prins et al., 2004).

On the other hand, a number of more recent researches have introduced quantitative methods of measuring WMH severity using computer-based techniques to obtain volumetric measures of WMH burden. These methods vary from manual outlining techniques to fully-automatic WMH detection (Jack et al., 2001; Anbeek et al., 2004; Wen and Sachdev, 2004; Admiraal-Behloul et al., 2005; Wu et al., 2006; Gibson et al., 2010; Cerasa et al., 2012; Simões et al., 2013).

Manual outlining techniques are referred to as region-of-interest (ROI) methodologies, in which the tracer inspects the scan using visualization softwares and manually draws WMH areas. The volume of each region is then calculated from the section thickness and the number of voxels included in the traced area. The values of all sections are then added together to make a total WMH volume. Manual outlining procedures, although accurate, have several limitations such as labor intensiveness, time consume, subjectiveness, and error-proneness. In addition, manual detection suffers from intra-observer and inter-observer variability (Smith et al., 2002).

In recent years, major progresses have been made on the development of semi- or fully-automated segmentations of WMH. These techniques are based on computer algorithms developed to replace human eye, allowing the collection of quantitative volumetric data on WMH (Jack et al., 2001; Anbeek et al., 2004; Wen and Sachdev, 2004; Admiraal-Behloul et al., 2005; Wu et al., 2006; Gibson et al., 2010; Cerasa et al., 2012; Simões et al., 2013). These techniques are more objective and free from user bias compared to the visual WMH ratings (Payne et al., 2002).

However, such methods have different accuracy, computational speed, and complexity. The majority of these approaches relies on multimodal data, thus being based on different MRI sequences, including T2-w, proton density (PD), T1-weighted (T1-w), inversion recovery (IR) and, more widely, FLAIR images. Innovative procedures to detect WMH have been developed based on Markov random field model (Schwarz et al., 2009), k-nearest neighbor (Anbeek et al., 2004; Wen and Sachdev, 2004), and neural classification (Dyrby et al., 2008), which require training images with WMH labels. The segmentation accuracy of these methods depends on the representative training data that may be difficult to select due to the heterogeneous nature of WMH. Differently, Admiraal-Behloul et al. (2005) proposed a fuzzy inference system to classify the WMH based on both anatomical locations and intensity values from three different MR images (T2-w, PD, and FLAIR) without the need of training samples. Moreover, some methods automatically or semi-automatically segment the WMH relying exclusively on FLAIR images by defining a cut-off threshold on the images (Jack et al., 2001; Wu et al., 2006; Gibson et al., 2010; Simões et al., 2013). In this context, Jack et al. (2001) and Gibson et al. (2010) employed empirical thresholds before applying linear fitting or fuzzy clustering to segment WMH. Similarly, Wen and Sachdev (2004) used the mean and standard deviation (SD) of gray matter (GM), white matter (WM), and cerebrospinal fluid (CSF) intensities to estimate the threshold for WMH and they used a WM probability map to identify the most likely WM regions. Recently, Sim o (2013) proposed a WMH segmentation method based on a modified context-sensitive Gaussian mixture model to determine voxel class probabilities, followed by a false positive correction step, where common FLAIR artifacts are eliminated from the segmentation.

All the quantitative methods here discussed have some limitations. Indeed, complex automatic or semi-automatic computer-based segmentation procedures require a large amount of technical resources that may not be available in clinical settings. Furthermore, multi-spectral approaches are not always accessible in clinical practice since the acquisition of all these images is cost-intensive and requires long processing time (Shen et al., 2008). Conversely, FLAIR-based approaches may, in sporadic cases, overestimate the WMH load due to FLAIR typical high intensity appearance in cortical areas, such as the septum pellucidum, and low artifacts in the fourth ventricle where a large percentage of false positive is detected (Wang et al., 2012). Such limitations make these methods scarcely applicable in large clinical contexts and barely replicable across sites.

In this study, we present a semi-automated method for WMH load measurement that overcomes the above presented limits. We applied the method on a group of MCI patients with various WMH load. The aims of our study are: (i) to develop a semi-automated algorithm for the detection, quantification, and localization of WMH using only FLAIR images and T1-w at 3T which is fully reproducible in different research contexts and clinical populations; and (ii) to evaluate the accuracy of the method by comparing it with manual segmentation.

## MATERIALS AND METHODS

### SUBJECTS

We recruited in our memory clinic in Rome, Italy, 30 patients with amnestic mild cognitive impairment (a-MCI; 21 males, 9 females; mean age ± SD = 72 ± 6 years, range 56–85; mean education = 9 ± 4 years; mean mini mental state examination (MMSE; Folstein et al., 1975; score = 26 ± 2) with a variable degree of WMH load. Specific inclusion criteria for a-MCI were (1) diagnostic evidence of a-MCI consistent with Petersen guidelines (Petersen et al., 1999), i.e., (i) complaint of defective memory, (ii) normal activities of daily living, (iii) abnormal memory function for age, and (iv) absence of dementia; and (2) suitability for MRI scanning.

Exclusion criteria were (1) major medical illnesses and autoimmune-inflammatory disease; (2) co-morbidity of primary psychiatric or neurological disorders, and (3) any other significant mental or neurological disorder. Medical and psychiatric histories were obtained from each subject, and all patients underwent a series of standard clinical examinations, including physical, neurological and mental status examinations, neurocognitive tests, and brain MRI (described below). All patients underwent the first diagnostic assessment and none were taking acetylcholinesterase inhibitors or psychotropic drugs (antidepressants, benzodiazepines, and antipsychotics). The study was approved and undertaken in accordance with the guidance of our local Ethics Committee and written consent was obtained from all participants.

### MRI ACQUISITION

All 30 participants underwent the same imaging protocol, which included 2D FLAIR and whole-brain high-resolution T1-w images, using a 3T Allegra MR imager (Siemens, Erlangen, Germany) with a standard quadrature head coil. All planar sequences acquisitions were obtained in the plane of the AC–PC line. Particular care was taken to center the subjects in the head coil and to restrain their movements with cushions and adhesive medical tape.

Two-dimensional FLAIR images were obtained in the axial plain (TE = 109 ms, TR = 8500 ms, slice thickness = 5 mm, slices = 24, matrix = 188 × 256, phase FOV = 0.73). Whole-brain T1-w images were obtained in the sagittal plane using a modified driven equilibrium Fourier transform (MDEFT, TE = 2.4 ms, TR = 7.92 ms, flip angle = 15°, voxel-size = 1 mm × 1 mm × 1 mm). All scans were visually inspected by a neuroradiologist (GL) with high expertise in clinical neuroimaging in order to exclude images with poor quality and motion artifacts.

**FIGURE 1:**
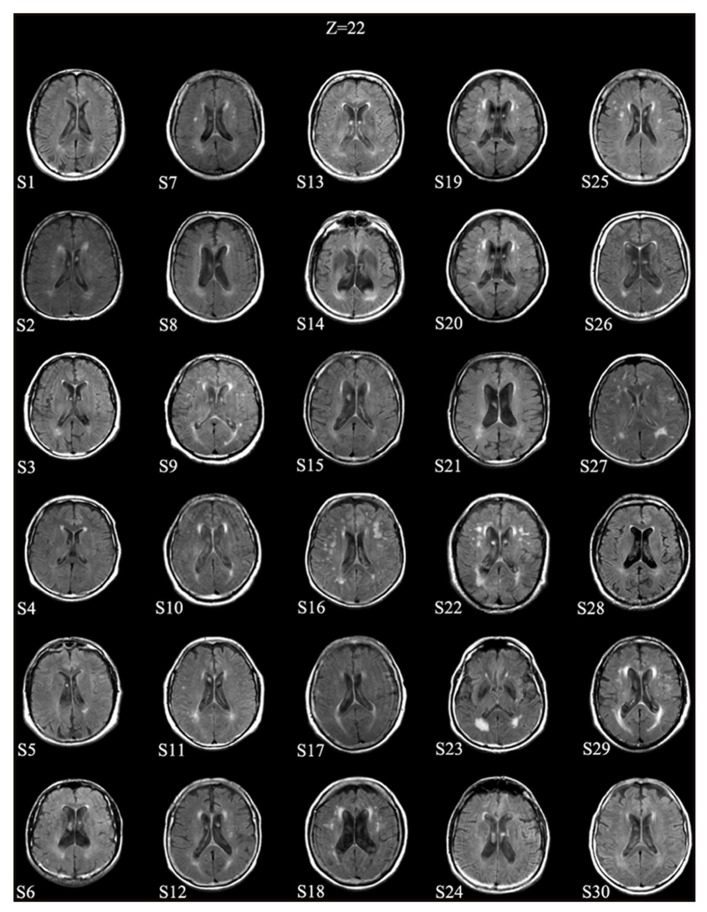
**Patients’ MRI scans.** A representative axial slice (at approximate MNI *Z*-level of 22) of FLAIR images for each patient is shown. Images are in neurological convention (left is left).

A representative axial slice of FLAIR images for each patient is shown in **Figure 1**.

### SEMI-AUTOMATED WMH SEGMENTATION

The semi-automated WMH segmentation algorithm was performed using FSL 4.1^1^ and the VBM8 toolbox^2^ implemented in SPM 8^3^ running in Matlab 2007b (MathWorks, Natick, MA, USA) and it consisted of three major steps:

(1) Image pre-processing, including non-brain tissue removal, spatial normalization into MNI space, removal of cerebellum and brain stem, and spatial filtering;
(2) Automated detection of WMH in FLAIR images and, if necessary, subsequent manual editing for removal of false positives;
(3) Post-processing, including the generation of WMH map and volumetric estimation of the WMH load at the individual and sample levels.

### STEP 1: PRE-PROCESSING

Image preprocessing included skull-stripping of the FLAIR images to restrict our analyses to brain tissue only reducing the risk of false positives and to improve the accuracy and efficiency of WMH segmentation.

#### Removal of non-brain tissues

For each subject, the FLAIR image was co-registered to the T1-weighted image using the FLIRT tool, integrated within FSL software through full affine alignment with nearest-neighbor resampling (correlation ratio cost function). Then, VBM8 with standard options was used to segment T1-weighted images and obtain a whole binary brain mask by summing the segmented GM, WM, and CSF in native space. The mask was then multiplied by the co-registered FLAIR and T1-w images to remove non-brain tissue. The average time for this step was 15 min per subject.

#### Spatial normalization of the skull-stripped FLAIR images into MNI space

The T1-w skull-stripped images were then linearly registered to MNI152 T1 1 mm brain template, and the transformation matrix was applied to the skull-stripped FLAIR images. Average time to complete the task for each subject was 1 min.

#### Removal of cerebellum and brain stem

Since WMH are rare in cerebellum and brain stem (Wen and Sachdev, 2004), we created a mask of these brain regions in MNI space to exclude them from the analysis. The mask was created using the Harvard–Oxford subcortical and the MNI structural atlases implemented in FSL 4.1. For each subject, the mask was then multiplied for the skull-stripped and normalized FLAIR image. The time to complete the task for each subject was negligible. In order to remove residual inhomogeneities, the normalized, skull-stripped FLAIR images were smoothed with a 2-mm full width at half maximum Gaussian kernel (Ashburner and Friston, 2000). This procedure was performed using FSL fslmaths utility. The average time to complete the step for each subject was 1 min.

Step 1 was performed using an in-house shell script which automatically concatenates all three substeps for all subjects.

### STEP 2: DETECTION OF WMH ON FLAIR IMAGES

#### Automatic identification of WMH on the intensity histogram of FLAIR images

For each subject we used FSL fslstats utility to calculate the mean and SD of the intensity of all brain voxels. In our procedure, after several trials, we optimized the process by choosing as threshold value the intensity mean +1.5 SD. This threshold was computed and applied to the FLAIR image for each subject, thus generating a map (of likely WMH) excluding all voxels having an intensity below such threshold. The time to complete the task was 1 min per subject and was implemented in a “for” cycle within a shell script.

#### Correction of false positives and negatives

**FIGURE 2:**
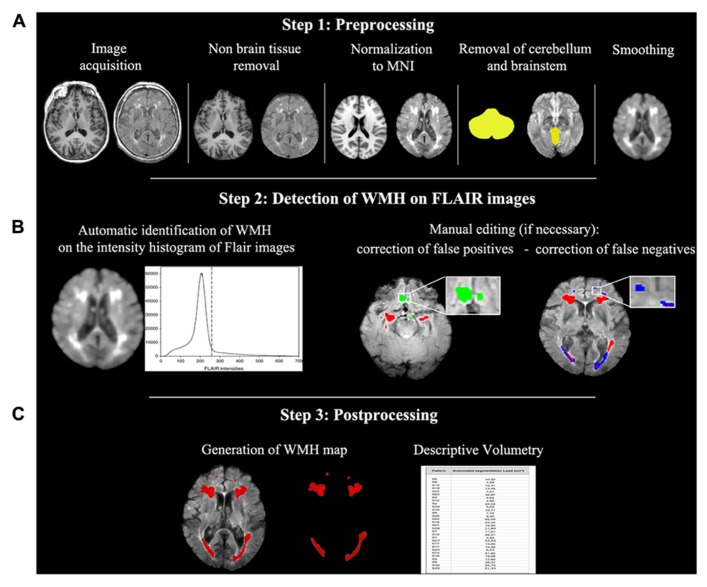
**Semi-automated WMH segmentation algorithm.** The process is represented including the preprocessing **(A),** the lesion detection **(B)** and the postprocessing **(C)** steps. Images are in neurological convention (left is left). MNI, Montreal Neurological Institute.

Each generated WMH map was visually inspected and compared to the original FLAIR image in order to identify false classifications (positive or negative). These were then manually corrected (as shown in **Figure 2**, middle row) by a trained neuropsychologist (FP) with a strong expertise in lesion analyses using the image processing software MRIcron^4^. In particular, wrong identifications of WMH were deleted through the *eraser* tool of MRIcron, while areas of WM lesions that were omitted by the algorithm were re-included. Correction of false positives was necessary for two patients while false negatives were present in nine patients (including the two were false positives were detected) and mainly consisted of underestimation of periventricular WMH volume. Manual correction required an average time of 8 min per patient.

### STEP 3: POST-PROCESSING

The final output provided by the system is a binary image in which a voxel is valued 1 if it is considered a WMH, 0 otherwise. Using these binary masks, for each subject, WMH volumes (expressed in cm^3^) were calculated automatically using FSL fslstats utility, again trough an automatic shell script developed in-house.

### MANUAL SEGMENTATION OF WMH

The manual segmentation of WMH on FLAIR images was performed by an expert neuroradiologist (Giacomo Luccichenti) and a trained clinician (Claudia Cacciari), expert in lesion segmentation, who were not aware of the results of the semi-automated procedure. Manual segmentation was delineated on the standard registered FLAIR images using MRIcron software by tracing the lesion outline with a mouse-controlled interface. This process resulted in the definition of binary images, considered as *gold standard.* For each subject, WMH volumes (expressed in cm^3^) were calculated automatically using FSL fslstats utility. The mean time to complete the task for each subject was 2 h and 32 min.

### STATISTICAL ANALYSES

Statistical analyses were performed with Statview software. The inter-rater reliability was calculated using the Spearman correlation coefficient. The differences between volumetric data derived from semi-automated and manual segmentations were evaluated using Student’s *t*-test while a linear regression model was computed to assess the relationship between the two datasets.

Furthermore, we assessed the similarities between the two datasets in terms of both volumetric and spatial agreement by computing the Dice similarity coefficient (DSC; Dice, 1945), which is a measure of sets agreement, given by the formula:

(1) DSC(Seg,Ref)=2|Seg∩Ref|(|Seg|+|Ref|),

where Seg is the automatically segmented image and Ref is the manually segmented image. DSC reflects differences in locations more strongly than differences in size (Zijdenbos et al., 1994). Indeed, this formula represents the size of the union of the Seg and Ref sets divided by the average size of the two sets. The DSC is commonly used when comparing datasets and, in particular, it has been widely used to assess similarities between automatic and manual segmentation procedures (Anbeek et al., 2004; Gibson et al., 2010; Cerasa et al., 2012; Simões et al., 2013). Generally, a DSC value of 0 indicates no spatial overlap and a value of 1 indicates perfect spatial alignment. Usually, DSC values of 0.7 and higher suggest good agreement between two delineations (Bartko, 1991).

## RESULTS

The semi-automated procedure of WMH quantification was successfully completed in all cases. Hyperintense signals on FLAIR images were present in all subjects, but their extent and distribution varied considerably (mean volume = 18.63 ± 14.81 cm^3^). The mean (±SD) volume of WMH from the two manual raters was 16.74 (±13.89) cm^3^ and 19.5 (±16.29) cm^3^ per subject (see **Table 1**).

**Table 1:** Results of semi-automated and manual segmentation of white matter hyperintensities.

Patient	WMH segmentation load (cm^3^)		
	Manual rater 1	Manual rater 2	Semi-automated
S1	3.65	3.78	3.49
S2	26.38	26.43	26.95
S3	10.64	14.63	12.69
S4	5.38	5.25	4.65
S5	10.84	10.98	9.49
S6	7.97	6.73	7.72
S7	12.51	14.75	11.21
S8	7.54	7.75	7.28
S9	37.22	37.39	35.27
S10	10.52	12.57	10.71
S11	9.55	12.74	15.26
S12	27.53	33.27	25.83
S13	9.05	8.15	10.31
S14	14.32	16.92	17.64
S15	4.67	4.98	5.22
S16	20.87	22.53	19.69
S17	8.40	10.19	13.90
S18	29.22	37.54	43.35
S19	12.27	15.60	13.40
S20	6.09	8.67	9.35
S21	13.24	20.27	15.93
S22	43.11	63.30	62.49
S23	31.76	37.84	32.87
S24	7.99	8.42	7.51
S25	6.56	7.01	6.55
S26	19.12	22.67	26.73
S27	24.46	28.96	35.23
S28	9.02	11.27	11.89
S28	65.70	68.34	51.33
S30	6.62	5.98	5.02
**Mean**	16.74	19.50	18.63
**SD**	13.89	16.29	14.81

*
The second, the third and the fourth columns respectively show the white matter hyperintensities (WMH) volumes from the two manual labels and the semi-automated segmentation. SD, standard deviation.
*

The two raters performed similarly in manual lesion segmentation (*r* = 0.976, *p* < 0.0001). Further, no statistical differences were found in the comparison between semi-automated and manual rater 1 WMH segmentation volumes (*t*-value = -1.79, DF = 29, *p* = 0.839) as well as between semi-automated and manual rater 2 WMH segmentation volumes (*t*-value = 1.113, DF = 29, *p* = 0.2749).

**FIGURE 3:**
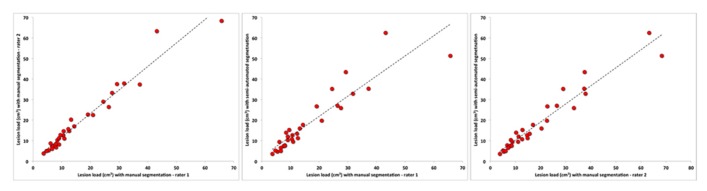
**Relationship between semi-automated and manual segmentation (rater 1 and rater 2).** Linear fits (dotted black line) are also reported.

Furthermore, as shown in **Figure 3**, the WMH volumes from the semi-automated segmentation method were highly correlated with volumes obtained through the manual method with a *r* = 0.921, *R*^2^ = 0.847, *F*_(1,29)_ = 155.540, *p* < 0.0001 for the manual rater 1 and *r* = 0.967, *r*^2^ = 0.935, *F*_(1,29)_ = 402.709, *p* < 0.0001 for the manual rater 2.

Finally, the two WMH segmentation procedures showed a very strong spatial similarity, with high DSC (manual rater 1 mean = 0.78, SD = 0.10; manual rater 2 mean = 0.77, SD = 0.14; see **Table 2**).

**Table 2:** Relationship between semi-automated and manual segmentation of white matter hyperintensities.

Patient	DSC		
	Manual rater 1Manual rater 2	Manual rater 1Semi-automated	Manual rater 2Semi-automated
S1	0.83	0.81	0.96
S2	0.79	0.70	0.70
S3	0.84	0.88	0.76
S4	0.74	0.66	0.64
S5	0.75	0.71	0.93
S6	0.72	0.70	0.85
S7	0.74	0.73	0.67
S8	0.75	0.74	0.97
S9	0.97	0.93	0.96
S10	0.80	0.67	0.59
S11	0.83	0.74	0.66
S12	0.73	0.68	0.87
S13	0.71	0.67	0.85
S14	0.72	0.70	0.68
S15	0.79	0.79	0.93
S16	0.91	0.94	0.93
S17	0.84	0.70	0.63
S18	0.82	0.71	0.61
S19	0.85	0.91	0.83
S20	0.78	0.72	0.61
S21	0.74	0.84	0.66
S22	0.80	0.80	0.67
S23	0.89	0.95	0.91
S24	0.95	0.96	0.94
S25	0.92	0.92	0.89
S26	0.87	0.79	0.71
S27	0.78	0.68	0.59
S28	0.87	0.84	0.76
S28	0.81	0.78	0.74
S30	0.70	0.71	0.57
**Mean**	0.81	0.78	0.77
**SD**	0.07	0.10	0.14

*The second column shows the Dice similarity coefficient (DSC) between two manual labels. The third and the fourth columns respectively show the DSC between the manual rater 1 and the semi-automated segmentation as well as between the manual rater 2 and the semi-automated segmentation. SD, standard deviation.*

## DISCUSSION

In the present study, we showed our semi-automated procedure for the detection, localization, and quantification of WMH on FLAIR images applicable to a wide range of patients with various diseases. This procedure is based on FLAIR and T1-w images (the latter are used for preprocessing purposes only, see **Figure 2**).

Results indicate that the algorithm performed remarkably well, compared to the gold-standard (manual segmentation by experts), since no statistical differences between the two outputs were found and a very high similarity emerged, both in terms of volumetric load and spatial location. This is an outstanding outcome, since the semi-automated procedure requires a time consumption which is approximately six times lower than the manual approach.

Other automated procedures developed to classify and quantify WMH have used a variety of classification approaches, including Markov random field model (Schwarz et al., 2009), k-nearest neighbor (Anbeek et al., 2004; Wen and Sachdev, 2004), neural classification (Dyrby et al., 2008), modified Gaussian mixture model (GMM) that incorporates neighborhood information (Simões et al., 2013) and threshold cut-offs (Jack et al., 2001; Gibson et al., 2010). Otherwise, our approach combines conservative voxel intensity thresholding with several key components that need further discussion.

First, we incorporated specific steps without any human intervention including the removal of non-brain tissue and of areas where WMH are unlikely (i.e., the cerebellum and the brainstem) in order to improve the accuracy of the method and overcome the limitations of subjectivity of the raters, thus ensuring test–retest reliability. Furthermore, these steps, which are not included in the majority of other methodologies, reduce the probability to include false positives and decrease the time spent to remove them, thus resulting in faster processing and more accurate maps of WMH.

Second, the transformation of FLAIR images from native to standard space (MNI), allows later statistical group comparisons and/or voxel-based statistics since the WMH images are in a common coordinates system. This feature enables a quantitative estimation and localization of WMH that can be further processed using statistical voxel-based image analysis softwares, which may be of importance for the study of the consequences of WM changes on cognition and behavior (Soderlund et al., 2006; Cacciari et al., 2010; Spalletta et al., 2013). Indeed, many studies suggested that the clinical significance of WMH is related not only to their volume but also to their localization (Chen et al., 2006). Indeed, since the distribution of WMH in specific functional regions may be closely related to specific types of cognitive impairment, clinical symptoms may be linked to the regional distribution of WMH. Moreover, an important implication of this automated procedure is its implementation in follow-up studies with repeated MRI scans. Allowing coregistration among different MRI sessions, our method will facilitate a more precise detection of eventual new WMH occurring at follow up, as well as of differences in their size and localization. This, particularly, would help in generating models of disease progression.

Moreover, our automated approach offers the possibility of a fully streamlined methodology with little user intervention and utilizes freely available software (e.g., FSL and SPM), which can be easily implemented on most desktop computers of standard power and are of routine use in most laboratories equipped to carry out neuroimaging research. This makes our approach reasonably accessible. Relatively little image manipulation is required and the quantification approach is procedurally straightforward. Additionally, little knowledge in computer programming allows to automate the steps and greatly reduce the processing time using in-house shell scripts which automatically concatenate all steps for all subjects.

A possible limitation of the presented methodology lies in the low number of brain slices used as inputs. In fact, the analyzed FLAIR images were acquired with a slice thickness of 5 mm which may be a poor resolution for accurate volumetric quantification and might induce interpolation errors in the registration processes. The MRI scanner used in the present study is relatively old and does not allow fast high-resolution 3D FLAIR imaging. A higher image resolution could improve the WMH quantification, and the registration accuracy, which would lead to more precise WMH localization; however, it would lead to an increase of acquisition time thus decreasing clinical practicability. However, modern MRI systems allow fast 3D FLAIR acquisition which would surely improve image resolution and, in turn, WMH segmentation.

In summary, we presented an automated detection and segmentation approach for WMH using T1-w and FLAIR images. By evaluating our method, we demonstrated that this segmentation approach is robust and reliable and we suggest that it has potential use in clinical studies.

Future works should consider the presented method to map cognitive consequences of WMH and their progression in time through large (and possibly multicenter) cross-sectional and longitudinal studies.

## Conflict of Interest Statement

The authors declare that the research was conducted in the absence of any commercial or financial relationships that could be construed as a potential conflict of interest.

## Author Contributions

Mariangela Iorio designed the study, analyzed imaging data, contributed to the writing of the manuscript; Gianfranco Spalletta designed the study, analyzed data, and contributed to the writing of the manuscript; Chiara Chiapponi analyzed imaging data and contributed to the writing of the manuscript; Giacomo Luccichenti analyzed imaging data and contributed to the writing of the manuscript; Claudia Cacciari analyzed imaging data; Maria Donata Orfei wrote the draft of the manuscript; Carlo Caltagirone contributed to the writing of the manuscript; Fabrizio Piras designed the study, analyzed imaging data, contributed to the writing of the manuscript.

## Footnotes

1

2

3

4
